# Serum IL-6 and IL-10 concentrations in bitches with pyometra undergoing ovariohysterectomy

**DOI:** 10.1186/s13028-015-0153-8

**Published:** 2015-09-26

**Authors:** Roman Dąbrowski, Josep Pastor, Marek Szczubiał, Tomasz Piech, Mariola Bochniarz, Władysław Wawron, Asta Tvarijonaviciute

**Affiliations:** Department and Clinic of Animal Reproduction, Faculty of Veterinary Medicine, University of Life Sciences in Lublin, 30 Gleboka St., 20-612 Lublin, Poland; Department of Animal Medicine and Surgery, Faculty of Veterinary Medicine, Universidad Autónoma de Barcelona, Barcelona, Spain; Department of Animal Medicine and Surgery, Faculty of Veterinary Medicine, Universidad de Murcia, Murcia, Spain

**Keywords:** Cytokines, Bitches, Pyometra, Ovariohysterectomy, Post surgery monitoring

## Abstract

**Background:**

Pyometra is a serious bacterial infection of the uterus affecting female dogs and manifests as an accumulation of pus in the uterine lumen. The aim of the study was to assess changes in serum interleukin (IL)-6 and IL-10 concentrations in bitches with pyometra undergoing ovariohysterectomy.

**Findings:**

Blood samples were collected from healthy bitches (controls) and bitches with pyometra before surgery, and 3 and 10 days after ovariohysterectomy. Before surgery, bitches with pyometra had significantly higher serum concentrations of IL-6 and IL-10 than the controls. After surgery, the serum concentration of IL-6 and IL-10 decreased significantly. In healthy dogs, the concentration of IL-6 and IL-10 showed a significant increase 3 days after surgery followed by a decrease on day 10.

**Conclusion:**

An increase in serum concentrations of IL-6 and IL-10 was present before surgery in bitches with pyometra and 3 days after ovariohysterectomy in healthy controls. Concentrations decreased after ovariohysterectomy and/or proper healing, suggesting that these cytokines can be useful for assessment of the postoperative period in bitches.

## Findings

Pyometra is an infection by opportunistic bacteria in the uterus resulting in inflammation and accumulation of pus. The condition may progress into sepsis [1, 2]. During inflammation, the acute phase response is activated [3], and its kinetics is predominantly affected by cytokines [4]. The production and release of pro-inflammatory cytokines is accompanied by the synthesis of anti-inflammatory cytokines thus creating a balance, which is essential for maintenance of homeostasis [5].

In dogs, interleukin 6 (IL-6) is one of the main pro-inflammatory cytokines, which is the key indicator of the biosynthesis and glycosylation of acute phase proteins (APPs) in the liver [6] and elevated concentrations reflect the activation of an inflammatory response [7]. In contrast interleukin 10 (IL-10) has been shown to inhibit cytotoxic effects of monocytes and macrophages as well as synthesis of pro-inflammatory cytokines and APPs. Because of this, IL-10 is recognized as a major suppressor of the immune response and inflammatory activity [8]. In human medicine, it has been reported that imbalance between IL-6 and IL-10 could contribute to the development of multiple organ dysfunction [4]. Furthermore, persistently high concentrations of IL-6 and/or IL-10, were associated with negative outcome of sepsis in humans [7]. Thus we hypothesized that in cases of canine pyometra alterations in IL-6 and/or IL-10 could be present. For this reason, the aim of the study was to assess concentrations of serum IL-6 and IL-10 in bitches with pyometra undergoing ovariohysterectomy (OHE) and compare these finding to healthy bitches undergoing elective OHE.

Dogs with pyometra consisted of 16 bitches (three Bull Terriers, four Golden Retrievers, four Dobermann Pinschers, two Boxers, one Shetland Sheepdog, and two mixed breed animals), aged 6–10 years, body weight (BW) 14–30 kg (mean BW 19.2 ± 5.8 kg) and body condition score (BCS) evaluated according to a 5-point scale [9] of 3. The dogs were admitted to the Department and Clinic of Animal Reproduction, University of Life Sciences, Lublin, Poland, for the surgical treatment of pyometra, i.e. OHE. Briefly, preliminary diagnosis of pyometra and therefore surgical treatment (OHE) was based on clinical examination and supported by laboratory tests. Clinical examination revealed polydipsia (75 %), polyuria (75 %), reduced or complete lack of appetite (87.5 %), dehydration (61.3 %), apathy (75 %), vomiting (31 %) and abnormal color of the mucous membranes (75 %). Additionally, a purulent vaginal discharge was present in 68.8 % of the bitches. An abdominal ultrasound examination revealed an enlarged uterus with a diameter ranging from 3 to 7 cm with hypoechogenic contents. Pyometra was confirmed by postoperative pathological examination of the uterus and ovaries.

The control group comprised of 11 clinically healthy bitches of six different breeds including mixed breed dogs, aged 2–4 years, BW 5–15 kg (mean BW 10.9 ± 4.3 kg), and a BCS of 3 admitted for elective OHE (spaying). The bitches were in diestrus as determined by history data, clinical examination and cytological examination of vaginal smears.

Bitches of both groups had participated in an earlier study in which we studied insulin-like growth factor I, C-reactive protein, and tryptophan [10, 11]. All experimental protocols and procedures were approved by the First Local Ethics Commission for Animal Experiments in Lublin, Poland.

Blood samples were collected from *vena cephalica* into silicone tubes with clot activator (VacutestKimas.r.l., Piove di Sacco PD, Italy) before surgery, and 3 and 10 days OHE. The blood were left to clot at room temperature for 30 min, and then centrifuged (3500 rpm, 5 min, 20 °C). The serum was then transferred to Eppendorf tubes. Serum was frozen at −80 °C until analysis. All samples were thawed on the same day within 5 months of the first sampling date and analysed.

IL-6 and IL-10 were determined using immunoassay kits (USCN Life Science Inc., Houston, USA) according to the manufacturer’s instructions [12, 13]. The absorbance was measured on a microtiter plate reader (ELx800, BioTek Instruments, USA) at 450 nm using 630 nm as reference. The assays were performed at the Independent Medical Biology Unit, Medical University of Lublin, Poland. Intra- and inter-assay coefficients of variation were below 10 and 12 %, respectively. The detection limit of canine IL-6 and IL-10 were 5.8 and 5.9 pg/ml as reported by manufacturer.

All data are presented as median (interquartile range) unless otherwise stated. D’Agostino and Pearson omnibus normality test was used to assess normality. To evaluate possible differences in baseline data between the two groups, Students t test was used for IL-10 data and Mann–Whitney test for IL-6 data. Within the group, one-way ANOVA test for repeated measures with Holm-Sidak’s multiple comparisons post-test was used with variables that were normally distributed and Friedman test with Dunn’s post-test in those that were not-normally distributed. Correlations between variables were determined using Spearman correlation analysis. Values of *P* < 0.05 for two sided analysis were considered significant. Statistical procedures were performed using Graph Pad Prism software (Graph Pad Prism Version 5 for Windows, Graph Pad software).

**Fig. 1:**
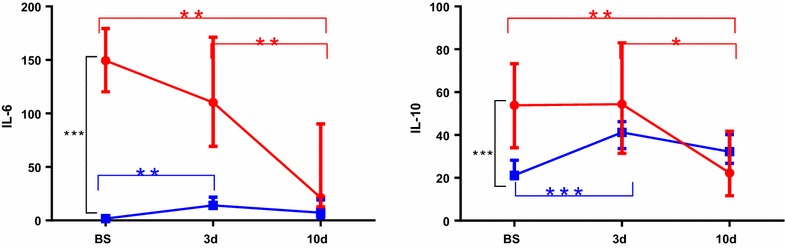
Median (interquartile range) of IL-6 and IL-10 in healthy dogs (*filled square*; n = 11) and dogs with pyometra (*filled circle*; n = 16) before surgery (BS), and 3 (3d) and 10 (10d) days after surgery

Before surgery, bitches with pyometra had higher serum concentrations of IL-6 and IL-10 (149.4 (120.2–179.5) pg/ml and 53.9 (34.1–73.3) pg/ml, respectively) than the controls [1.8 (1.8–2.0) pg/ml and 21.2 (19.5–28.2) pg/ml] (*P* < 0.001 for both IL-6 and IL10) (Fig. 1).

Compared to the presurgery concentration bitches with pyometra had decreased serum concentration of IL-6 and IL-10 3 days after OHE (*P* < 0.01 and *P* < 0.05, respectively) and 10 days after OHE (*P* < 0.01 for both IL6 and IL10) (Fig. 1). Healthy dogs showed an increase in IL-6 and IL-10 levels 3 days after surgery (*P* < 0.05 and *P* < 0.01, respectively) followed by a decrease on day 10 after OHE (Fig. 1).

When all data were pooled, a statistically significant positive correlation was observed between IL-6 and IL-10 levels (r = 0.519; *P* < 0.001).

Pyometra is an infection linked with systemic inflammation with multiple organ dysfunction and increased mortality rates in bitches [1, 14–16]. During the inflammatory process, the acute phase response is activated [17], which leads to changes in biosynthesis of certain pro- and anti-inflammatory cytokines [8, 14] as seen in our study with increased presurgery levels of pro-inflammatory IL-6 and anti-inflammatory IL-10. In pyometra, the primary immune response is mediated by e.g. IL-6, the concentrations of which is both delayed and sustained in comparison with other cytokines [14]. A high concentration of IL-6 in bitches with pyometra indicates an activation of the cellular immune system [14]. Induction of cytokine synthesis during the course of pyometra may result from an influence of endotoxin—lipopolysaccharide present in the cell walls of Gram-negative bacteria such as *Escherichia coli,* the most commonly isolated bacteria from cases of pyometra [1, 18]. Based on our result we suggest, that production of IL-6 and IL-10 is upregulated in bitches having pyometra. These data, however, partly differ from previously studies [8, 19]. In one study, an increase in IL-10, but not in IL-6 was detected [19] while a study by Kjelgaard-Hansen et al. [8] found no significant differences in serum IL-10 concentration between dogs with and without pyometra. These disagreements could be due to mainly two reasons. First, the methods for IL analysis differ and may not be sensitive enough to detect low concentrations. This may be the cause in the study by Karlsson et al. [19], who detected IL-6 in only three dogs out of 32. The other reason could be the inclusion of relatively low number of animals together with high inter-individual variation, resulting in an absence of significant differences between the evaluated groups.

The novelty of our study includes the evaluation of IL-6 and IL-10 in bitches during the recovery period after OHE. The levels of IL6 and IL10 decreased significantly in the bitches with pyometra on days 3 and 10 after surgery as also reported by others [20]. In the group of bitches with elective OHE, the maximum mean levels of IL-6 and IL-10 were found 3 days after surgery, with a subsequent decrease on day 10, which indicates that the acute phase response caused by the surgical procedures is mediated by IL-6 [21] and is accompanied by an increase in IL-10. We hypothesize that a proper postoperative course depends largely on serum concentrations of these cytokines, irrespective of the factor inducing inflammation [22].

In conclusion, an increase in serum concentrations of IL-6 and IL-10 occurred before surgery in bitches with pyometra and 3 days after OHE in healthy control bitches undergoing elective OHE. Concentrations decreased after removal of the inflamed uterus and after proper healing, suggesting that these cytokines can be useful for assessment of the postoperative period in bitches.
